# A Case of Omental Bleeding as a Result of Segmental Arterial Mediolysis Treated Successfully by Laparoscopic Partial Omentectomy

**DOI:** 10.70352/scrj.cr.25-0262

**Published:** 2025-07-12

**Authors:** Yudai Mimata, Nobuhiko Kanaya, Yoshitaka Kondo, Hitoshi Minagi, Yoshihiko Kakiuchi, Shinji Kuroda, Kunitoshi Shigeyasu, Shunsuke Kagawa, Toshiyoshi Fujiwara

**Affiliations:** Department of Gastroenterological Surgery, Okayama University Graduate School of Medicine, Dentistry and Pharmaceutical Sciences, Okayama, Okayama, Japan

**Keywords:** segmental arterial mediolysis, laparoscopic partial omentectomy, hemoperitoneum

## Abstract

**INTRODUCTION:**

Segmental arterial mediolysis (SAM) is a rare, non-atherosclerotic, non-inflammatory arteriopathy characterized by lysis of the arterial media, leading to aneurysm formation and possible rupture. Although visceral arteries are typically involved, SAM-induced omental bleeding is extremely uncommon. While transcatheter arterial embolization (TAE) has been reported, surgical resection offers both definitive hemostasis and histopathological confirmation.

**CASE PRESENTATION:**

A 56-year-old man presented with upper abdominal pain without a history of trauma. Contrast-enhanced CT revealed a hematoma and fusiform dilation of an omental artery, suggesting omental hemorrhage. As he was hemodynamically stable, initial conservative management was chosen. However, a follow-up CT on day 7 demonstrated aneurysm enlargement, prompting laparoscopic partial omentectomy. Intraoperative findings included a 5-cm hematoma in the central omentum. Histopathological examination showed vacuolization of the tunica media and loss of the internal elastic lamina, confirming the diagnosis of SAM. The patient had an uneventful postoperative course and was discharged on the 3rd postoperative day.

**CONCLUSIONS:**

This rare case of SAM-related omental bleeding was successfully treated with laparoscopic partial omentectomy. Tailored treatment strategies including laparoscopic surgery are essential for optimal outcomes in SAM.

## Abbreviations

MS
: multiple sclerosis
SAM
: segmental arterial mediolysis
SMA
: superior mesenteric artery
TAE
: transcatheter arterial embolization

## INTRODUCTION

Spontaneous rupture of omental vessels is an uncommon but potentially life-threatening condition that can result in severe intra-abdominal hemorrhage. Despite its rich vascularity, primary omental bleeding is rare and can present with nonspecific symptoms, often leading to diagnostic challenges.^1)^

Omental bleeding may arise from a variety of causes, including trauma, neoplasia, arterial aneurysm rupture, and vasculitis.^2–5)^ However, in some cases, no apparent cause can be identified, leading to the classification of idiopathic omental bleeding.^1)^ Among the rare vascular pathologies affecting the omental arteries, SAM has been recognized as a distinct entity. First described by Slavin and Gonzalez-Vitale, SAM is a non-inflammatory, non-atherosclerotic arteriopathy characterized by segmental destruction of the arterial media, leading to aneurysm formation, arterial dissection, and potential rupture. While SAM can involve various visceral arteries, its presentation as an isolated cause of omental hemorrhage is exceedingly rare.^6)^

Here, we report a rare case of omental bleeding due to SAM that was successfully treated with laparoscopic partial omentectomy. This case highlights the importance of considering SAM in the differential diagnosis of spontaneous omental hemorrhage and underscores the role of laparoscopic surgery as a definitive treatment strategy.

## CASE PRESENTATION

**Fig. 1:**
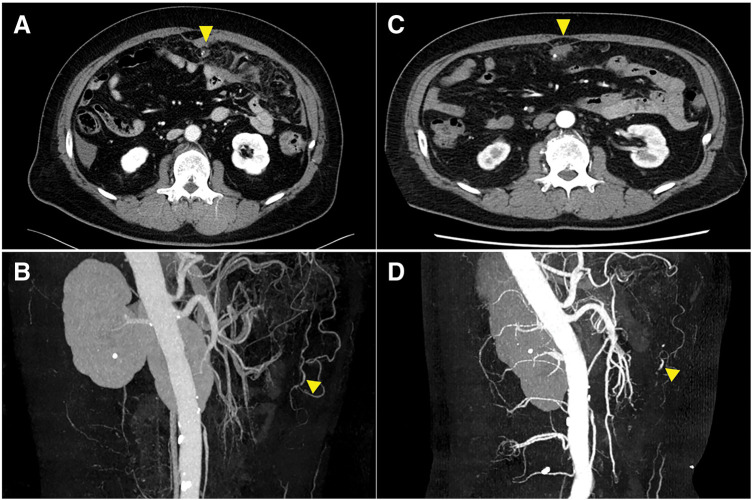
Images of contrast-enhanced CT. (**A**, **B**) Contrast-enhanced CT revealed a hematoma and an aneurysm in the omentum (triangle). (**C**, **D**) The aneurysm has grown slightly in size in 7 days (triangle).

A 56-year-old man presented to the emergency department complaining of upper abdominal pain. He had no history of abdominal trauma. His past medical history was significant for multiple sclerosis, for which he was taking fingolimod hydrochloride. On physical examination, his blood pressure was 123/81 mmHg and his pulse rate was 90 beats/min. Tenderness was noted throughout the abdomen, and his abdomen was rigid. Laboratory results showed a hemoglobin level of 12.6 g/dL. Contrast-enhanced CT revealed a high-density area in the midline from the umbilical to the lower abdominal region, and a fusiform dilation was observed at the distal end of an omental branch artery (**Fig. 1A**, **1B**). Omental hemorrhage was suspected; however, due to stable vital signs and the absence of significant progressive anemia, a conservative management strategy was adopted. A follow-up CT scan 7 days later showed enlargement of the aneurysm, and there was no improvement in the ascites located at rectovesical fossa, which prompted a laparoscopic partial omentectomy (**Fig. 1C**, **1D**).

**Fig. 2:**
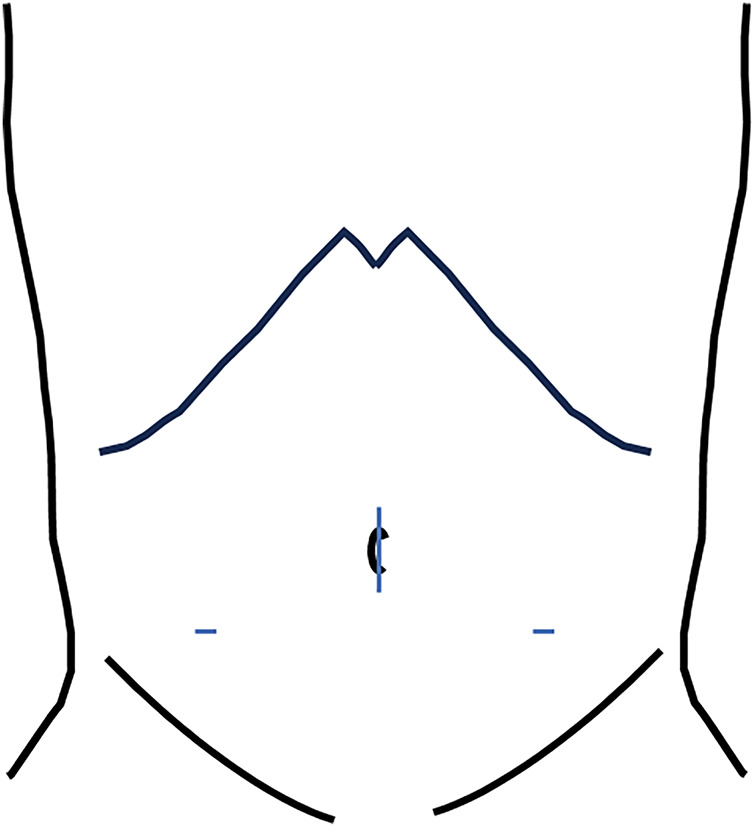
Size and distribution of trocars. Umbilical region: EZ-access device; bilateral lower abdomen: 5 mm.

**Fig. 3:**
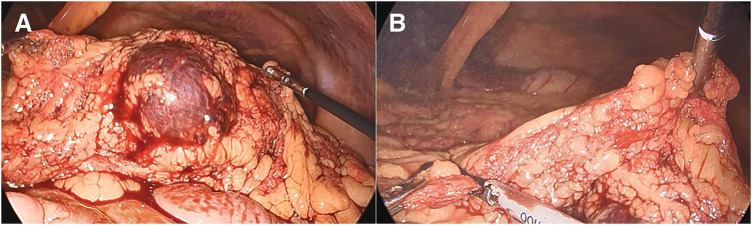
Intraoperative images. (**A**) Hematoma was identified in the central portion of the omentum. (**B**) A part of the omentum was removed from both sides of the hematoma.

**Fig. 4:**
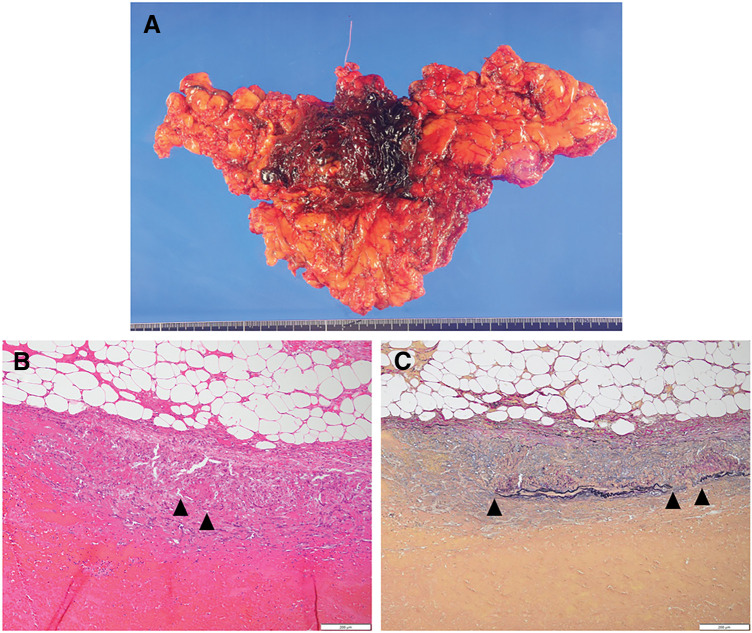
Pathological imaging of resected omentum. (**A**) Resected omentum. (**B**) Hematoxylin and eosin staining shows the vacuolization of smooth muscle cells of the tunica media (triangle). (**C**) Elastica van Gieson staining reveals that the disappearance of internal elastic lamina was observed at the site of tunica intima rupture (triangle).

The sizes and distributions of trocars are shown in **Fig. 2**. We observed bloody ascites in the pelvic cavity and bilateral paracolic gutters. First, the omentum, including the hematoma, was found to be adhered to the cephalad side, and this was dissected (**Fig. 3A**, **3B**). A 5-cm hematoma was identified in the central portion of the omentum near the transverse colon. The omentum was transected by an ultrasonically activated device from the left side toward its attachment to the transverse mesocolon. It was similarly transected from the right side, and the resected specimen was removed. The total size of the resected omentum was 6.0 × 4.5 cm (**Fig. 4A**). The operative time was 112 min and the estimated blood loss was 120 mL. Pathological examination of the resected omentum revealed loss of the media and formation of granulation tissue in the arterial wall, with vacuolization observed in the remaining media (**Fig. 4B**, **4C**). Based on these findings, the diagnosis of omental hemorrhage due to SAM was made. The patient was discharged on the 3rd postoperative day. No signs of recurrence have been observed since.

## DISCUSSION

Omental bleeding, although relatively rare, can result in life-threatening hemorrhagic shock due to massive intra-abdominal hemorrhage. In our case, a laparoscopic partial omentectomy was performed for an enlarging aneurysm, which was rarely caused by SAM.

Within the spectrum of aneurysmal ruptures, Slavin and Gonzalez-Vitale introduced the concept of SAM.^7)^ SAM is a distinct vascular pathology characterized by segmental dissolution of the media of abdominal visceral arteries, frequently resulting in the formation and subsequent rupture of aneurysms. SAM most commonly affects the splanchnic arteries, particularly the branches of the SMA, celiac artery, and less frequently, the renal and intracranial arteries. The etiology of SAM remains unclear. Proposed mechanisms include vasospasm triggered by catecholamines, endothelial dysfunction, and inflammatory responses. Interestingly, our patient had a history of MS and was receiving fingolimod, an S1P receptor modulator that influences vascular integrity and immune function.^8)^ While no direct association between MS and SAM has been established, prior reports suggest that MS patients might have a higher prevalence of vascular abnormalities, including aneurysm formation.^9)^ Immune dysregulation and chronic inflammation in MS might contribute to vascular wall fragility, predisposing patients to conditions such as SAM. Further research is needed to elucidate potential links between MS, immunomodulatory therapies, and arterial pathology.

In recent years, TAE has emerged as a potential treatment modality for SAM, with increasing case reports documenting successful outcomes.^10,11)^ In cases presenting with hemorrhagic shock, urgent angiography should be performed after initial resuscitation with fluids and vasopressors, and endovascular intervention should be considered based on the patient’s condition and angiographic findings. However, cases of re-rupture after TAE have been reported,^12)^ underscoring the importance of recognizing the limitations of this approach. In addition, given that the arterial wall is prone to dissection and aneurysm formation from medial lysis, intraarterial catheter manipulation and balloon dilation may result in progression or development of new arterial dissections.^13)^ Furthermore, there have been reported cases where aneurysms worsened during observation without treatment.^6)^ In this case, we performed surgery because the aneurysm showed an enlargement and no improvement of ascites. Surgical intervention offers the advantage of direct visualization and pathological confirmation of the diagnosis, particularly in ruling out other vascular pathologies and malignancies. With advancements in laparoscopic techniques and instrumentation, laparoscopic surgery has become increasingly feasible and offers the potential benefits of reduced invasiveness. While open surgery remains the preferred approach in cases of massive intra-abdominal hemorrhage and hemodynamic instability, we believe that laparoscopic surgery can be a valuable option in patients with stable vitals. A tailored treatment approach, chosen on an individual basis, is essential to ensure the best possible outcome.

## CONCLUSIONS

We experienced a case on whom laparoscopic partial omentectomy was performed for omental hemorrhage, leading to a diagnosis of SAM. Laparoscopic surgery might be a useful option in stable patients. It is necessary to develop a treatment strategy tailored to each individual case.

## ACKNOWLEDGMENTS

We appreciate the support of the staff at the Department of Pathology, Okayama University Hospital, for providing pathological images.

## DECLARATIONS

### Funding

None.

### Authors’ contributions

Y.M. and N.K. contributed to the conceptualization and drafting of the report.

Y. Kondo, K.S., H.M., Y. Kakiuchi, S. Kuroda, and S. Kagawa were involved in patient management.

T.F. oversaw patient care and supervised the case.

All authors have reviewed and approved the manuscript and take full responsibility for its content.

### Availability of data and materials

All relevant datasets supporting the conclusions of this article are included within the manuscript.

### Ethics approval and consent to participate

This case report was approved by the Institutional Review Board of Okayama University Hospital (No. 2774). Written informed consent for treatment was obtained from the patient.

### Consent for publication

The patient provided written consent for publication of this case report.

### Competing interests

The authors declare no competing interests related to this article.
